# The Evolving Story of Autoantibodies in Pemphigus Vulgaris: Development of the “Super Compensation Hypothesis”

**DOI:** 10.3389/fmed.2018.00218

**Published:** 2018-08-14

**Authors:** Animesh A. Sinha, Thomas Sajda

**Affiliations:** Department of Dermatology, University at Buffalo, Buffalo, NY, United States

**Keywords:** autoantibodies, desmoglein, thyroid peroxidase, acetylcholine receptor, mitochondria, desmocollin, plakophilin, protein array technology

## Abstract

Emerging data and innovative technologies are re-shaping our understanding of the scope and specificity of the autoimmune response in Pemphigus vulgaris (PV), a prototypical humorally mediated autoimmune skin blistering disorder. Seminal studies identified the desmosomal proteins Desmoglein 3 and 1 (Dsg3 and Dsg1), cadherin family proteins which function to maintain cell adhesion, as the primary targets of pathogenic autoAbs. Consequently, pathogenesis in PV has primarily considered to be the result of anti-Dsg autoAbs alone. However, accumulating data suggesting that anti-Dsg autoAbs by themselves cannot adequately explain the loss of cell-cell adhesion seen in PV, nor account for the disease heterogeneity exhibited across PV patients has spurred the notion that additional autoAb specificities may contribute to disease. To investigate the role of non-Dsg autoAbs in PV, an increasing number of studies have attempted to characterize additional targets of PV autoAbs. The recent advent of protein microarray technology, which allows for the rapid, highly sensitive, and multiplexed assessment of autoAb specificity has facilitated the comprehensive classification of the scope and specificity of the autoAb response in PV. Such detailed deconstruction of the autoimmune response in PV, beyond simply tracking anti-Dsg autoAbs, has provided invaluable new insights concerning disease mechanisms and enhanced disease classification which could directly translate into superior tools for prognostics and clinical management, as well as the development of novel, disease specific treatments.

## Introduction

Pemphigus vulgaris (PV) is an autoimmune skin disease that results from the production of autoAbs that target keratinocyte proteins. Binding of these autoAbs results in the loss of keratinocyte cell-cell adhesion (termed acantholysis) just superior to the basal cell layer in the epidermis resulting in the development of painful, flaccid bullae on the skin and/or mucosal membranes that easily rupture. The discovery that autoantibodies (Abs) targeting desmoglein (Dsg) 1 and Dsg3 cause blister formation has been potentially the most critical event in understanding disease pathogenesis in PV to date. Numerous studies have been dedicated to characterizing the isotype and fine epitope specificity of anti-desmoglein autoAbs and investigations aimed at uncovering the mechanisms underlying autoAb-induced blister formation focused primarily on studying the effects downstream of anti-desmoglein Ab binding. Accordingly, the majority of currently proposed disease models are desmoglein-centric. These models, however, fail to explain a number of disease phenomena such as patients that present in active disease without detectable anti-desmoglein autoAbs and the lack of tight correlation between anti-desmoglein autoAb titers and disease activity. Additionally, these models cannot adequately account for the degree of disease heterogeneity exhibited by PV patients. Here, we focus on: (i) the seminal studies that led investigators to identify Dsg3 and 1 as targets of pathogenic autoAbs, and how these studies shaped our understanding of disease, and (ii) the identification of non-Dsg autoAbs, with a particular focus on the contribution of comprehensive autoAb profiling facilitated by protein microarray technology, as well as the potential role of these autoAbs in disease, and how these findings may re-shape/direct how we ultimately view the pathogenesis of PV.

## Early studies

**Figure 1:**
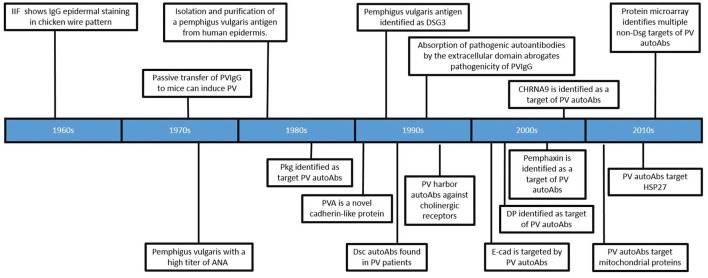
Timeline of significant findings regarding autoantibodies in Pemphigus vulgaris. Important developments in the field are depicted in chronological order (see text for detailed description).

Several observations have suggested a role for autoAbs in the pathogenesis of PV (Figure 1). Neonates born to mothers with PV were observed to experience transient disease at birth (1), and the addition of the IgG fraction alone from patient sera (PVIgG), without the presence of complement or other immune cells, could recapitulate disease in a skin organ culture model as well as disturb cell-cell adhesion in a keratinocyte monolayer (2, 3). Patient sera was shown to be capable of inducing disease when passively transferred to mice (4). Furthermore, PVIgG stained epidermal tissue in a “fishnet pattern.” Early efforts to determine the target of these autoAb revealed that PV sera recognized a number of then unknown proteins, with molecular weights of 20, 22, 33, 50, 66, 68, 80, 105, 130, 140, 160, 210, and 220 kDa (5–14). The effect of PV sera on cell adhesion led researchers to hypothesize, and eventually prove, that PV sera recognized a desmosomal protein (15, 16). In 1991, using PV sera to screen a phage display library created from cDNA cloned from normal human epidermal keratinocytes, Amagai et al. (6), demonstrated that the antigen recognized by PV autoAbs was a novel 130 kDa cadherin protein that shared a high degree of homology with desmoglein 1, a previously discovered desmosomal cadherin. Eventually this novel cadherin was named desmoglein 3. However, in order to identify this clone, only PV autoAbs purified from the 130 kDa band were used to screen the phage display library, because initial screening of the library with PV sera identified over 200 clones, and none of those 200 clones were capable of being recognized by all sera samples (6).

After the discovery of Dsg3 as a major antigenic target of PV autoAbs, a number of studies focused on establishing the pathogenicity of anti-Dsg3 autoAbs. In an early experiment, PVIgG was exposed to fusion proteins consisting of various Dsg3 extracellular domains (ECs) conjugated to beta galactosidase. PVIgG from 17 of 23 patients recognized at least one of the fusion proteins, but 6 patients did not possess autoAbs reactive to any of the constructs. Two different fusion proteins, expressing EC1-2 and EC3-5 respectively, were then used to deplete anti-Dsg3 autoAb from PVIgG. When autoAbs affinity purified by the EC1-2 construct were passively transferred to mice, they were capable of eliciting blister formation. However, instead of the gross blister formation seen when using PVIgG, these purified autoAbs only produced microscopic blisters. Additionally, PVIgG depleted using the EC1-2 construct was still capable of eliciting blister formation upon passive transfer. AutoAbs purified using the EC3-5 construct failed to induce blister formation upon passive transfer. From these experiments, the authors concluded that anti-Dsg3 autoAb are in fact disease causing, and the failure of the Dsg3 constructs to be recognized by all patient sera, as well inhibit the pathogenicity of PVIgG, was due to improper conformation of the Dsg3 constructs (17).

A different Dsg3 construct, consisting of the extracellular domains of Dsg3 combined with the constant region of IgG1 (named PVIg), was generated to create a protein that would more accurately reflect the conformation of native Dsg3. Preabsorption of PVIgG with PVIg reduced the indirect immunofluorescent titers of 6/17 patients to zero, significantly lowered the indirect immunofluorescent titers of all but one patient's IgG, which remain unaffected. Preabsorption of PVIgG with this construct was also capable of preventing the formation of gross blisters when transferred to mice, although microscopic suprabasilar acantholysis was still detected in some areas (18). It should be noted that the specificity of the autoAbs purified using this construct were not assessed in this experiment.

A number of mouse models also seemed to support the notion that anti-Dsg3 could be sufficient to cause disease in PV. Splenocytes from Dsg3^−/−^ mice immunized with Dsg3 were adoptively transferred to rag2^−/−^ mice which subsequently developed blisters and suprabasilar acantholysis (19–21). Histological examination of the epidermis from mice with a targeted deletion of Dsg3 displayed suprabasilar acantholysis as well as the characteristic “tombstoning” of basal keratinocytes as seen in PV, but still lacked any gross signs of spontaneous blister formation (22). In another experiment, mice expressing a truncated Dsg3 displayed swelled paws, flaky skin, widened intercellular space between keratinocytes and a number of other epidermal abnormalities. Again, blister formation and suprabasilar acantholysis were absent in these mice (23).

## The desmoglein compensation hypothesis

The identification of Dsg3 as a major antigenic target represented a critical moment in the process of understanding PV and, after its discovery, the prevailing view of disease immediately narrowed. For the next decade, the design and interpretation of almost all experiments were informed by an underlying assumption that anti-Dsg autoAb were the sole drivers of disease in pemphigus, ignoring the potential role of other, non-Dsg autoAbs. Although the importance of anti-Dsg3 autoAb is clear, this limited view may have slowed the progression of understanding the true complexity of disease. The impact of how this desmoglein-centric view fundamentally influenced the way researchers understood PV is epitomized by the development of the *desmoglein compensation hypothesis*. This elegant hypothesis asserts that anti-Dsg3 and anti-Dsg1 autoAb profiles can predict which epithelial surface(s) will be affected, as well at what level the loss of cell-cell adhesion will occur in the epidermis (24). The foundation of this hypothesis are the differential expression patterns of Dsg3 and Dsg1 between mucosal and cutaneous epidermis, and the idea that Dsg3 or Dsg1 alone can sustain cell-cell adhesion. In a series of experiments Mahoney et al. demonstrated that: (1) murine mucosal tissue expresses Dsg3 throughout the entire epidermis, with strongest expression in the superficial layers, while Dsg1 expression is highest in the superficial layers and very low in the deeper layers, and (2) murine cutaneous epidermal tissue expresses Dsg3 most highly in the basal layer with lower expression seen in the more superficial layers, whereas Dsg1 expression is high in superficial epidermis and decreased in the deeper layers. The tissue specific expression patterns of Dsg3 and 1 in mice are similar to that of human epidermis, with the exception that Dsg1 expression in human mucosal epidermis is very low (25–27).

Next, a series of passive transfer experiments using PVIgG injected into wildtype C57BL/6J mice or Dsg3 null mice demonstrated that anti-Dsg1 autoAbs induce blister formation when transferred to Dsg3 null mice and both anti-Dsg3 and anti-Dsg1 autoAb are required to promote blister formation in parts of the epidermis that express both Dsg3 and 1. It should be noted that the Dsg3 null mice used in this experiment were shown to spontaneously develop inflammatory erosions along with a loss of cell-cell adhesion in the superficial layers of the epidermis (22). Still, from these results, the authors proposed that blister formation in PV occurs initially within the mucosa at the suprabasilar level where Dsg3, but not Dsg1, is expressed. Cutaneous lesions in PV patients only occur when patients develop additional autoAbs, directed against Dsg1, later in disease. This hypothesis also attempts to explain blister formation in Pemphigus foliaceus (PF), a related autoimmune blistering disease where autoAbs directed against Dsg1 cause cutaneous blister formation in the superficial layers of the epidermis.

Aside from the assertion that the epidermal architecture of the Dsg3 null mice used in these experiments may not have been an optimal model, there exists a plethora of clinical and experimental evidence that does not align with the desmoglein compensation hypothesis. If true, PV patients exhibiting both anti-Dsg3 and Dsg1 autoAbs might be expected to demonstrate a loss of cell-cell adhesion throughout the entire epidermis, instead of just at the suprabasilar level. Moreover, assessment of Dsg3 and 1 titers in PV patients have demonstrated the existence of cutaneous only patients (with no history of mucosal lesions) with no detectable anti-Dsg1, mucosal only patients with no detectable anti-Dsg3, as well patients that lack detectable titers of anti-Dsg3 or 1 autoAbs (28–43), all of which cannot be explained by the desmoglein compensation hypothesis (Table 1).

**Table 1:** Postulates and limitations of the desmoglein compensation hypothesis (DHC).

**Postulates of the DCH**	**Limitation of the DCH**
- Patients expressing only anti-Dsg3 autoAbs exhibit suprabasal acantholysis in mucosal epidermis only.	- Patients expressing only anti-Dsg3 autoAbs can exhibit cutaneous acantholysis either alone or in combination with mucosal acantholysis.
- Patients expressing both anti-Dsg3 and anti-Dsg1 autoAbs will exhibit only suprabasal acantholysis in both mucosal and cutaneous epidermis.	- Patients with no detectable levels of anti-Dsg3 or anti-Dsg1 autoAbs can exhibit cutaneous acantholysis either alone or in combination with mucosal acantholysis.
- Patients expressing only anti-Dsg1 autoAbs will exhibit acantholysis in the superficial cutaneous epidermis only (PF).	- Patients expressing both anti-Dsg3 and anti-Dsg1 autoAbs can exhibit cutaneous lesions only, rather than cutaneous and mucosal lesions.
	- Patients expressing both anti-Dsg3 and anti-Dsg1 autoAbs exhibit only suprabasal acantholysis in both cutaneous and mucosal lesions.

## Non-desmoglein targets of autoantibodies in PV

The desmoglein compensation hypothesis cannot adequately account for disease presentation in PV, and newer models are needed to more precisely understand disease mechanisms. The idea that unique profiles of autoAbs may underlie differences in disease expression represents the beginning of a more sophisticated understanding of immune dysregulation in PV. The failure of anti-Dsg autoAbs alone to fully explain disease spurred the notion that additional autoAb specificities may be relevant in PV, and subsequent experiments have resulted in a growing pool of evidence that suggests autoAbs directed at *non-Dsg targets* may play a role in PV.

Initial evidence that non-Dsg autoAb may be relevant to disease came from experiments demonstrating the formation of blisters in Dsg3 null mice upon the passive transfer of PVIgG that did not contain any anti-Dsg1 autoAbs (44). Although this observation seemed to contradict previous studies that demonstrated the ability of a Dsg3 fusion protein to absorb out pathogenic antibodies in PVIgG, it was soon shown that autoAbs eluted from this protein bound to a number of distinct protein bands when exposed to the lysate of keratinocytes lacking expression of Dsg3 (45). Potentially, the ability of this construct to absorb out non-specific IgG is attributable to Fc-Fc interactions (46–48). Additional evidence that non-Dsg autoAbs may be relevant to disease came from studies that demonstrated a lack of correlation between anti-Dsg autoAb titers and disease activity in a subset of patients (29, 41, 42, 49, 50). These studies emphasized the importance of identifying other targets of autoAbs in PV, and soon more than 50 non-Dsg antigens were reported to be recognized by PV patient autoAbs (Table 2).

**Table 2:** Ranking evidence for non-desmoglein antigens.

**Level of evidence**	**Symbol**	**Name**	**IB/IP/ELISA**	**Protein microarray**	***In vitro***	***In vivo***	**References**
3	CHRNA9	Cholinergic Receptor Nicotinic Alpha 9			x	x	(51)
3	ANXA9	Annexin A9, Pemphaxin	x		x	x	(52)
3	DSC3	Desmocollin 3	x	x	x		(53–57)
3	TPO	Thyroid Peroxidase	x	x	x		(58–63)
2	CD2	T-cell surface antigen T11/Leu-5, LFA-2, LFA-3 receptor	x	x			(64)
2	CD33	Sialic acid binding Ig-like lectin 3	x	x			(55, 64)
2	CD36	Thrombospondin receptor	x	x			(64)
2	CD37	Cluster of Differentiation 37 Molecule, Leukocyte antigen 37	x	x			(64)
2	CD40	Cluster of Differentiation 40 Molecule	x	x			(64)
2	CD84	Cluster of Differentiation 84 Molecule	x	x			(64)
2	CEACAM6	Carcinoembryonic Antigen Related Cell Adhesion Molecule 6	x	x			(64)
2	CHRM1	Cholinergic Receptor Muscarinic 1	x	x			(64)
2	CHRM3	Cholinergic Receptor Muscarinic 3	x	x			(63, 65, 66)
2	CHRNA5	Cholinergic Receptor Muscarinic 5	x	x			(67)
2	CHRNA10	Cholinergic Receptor Nicotinic Alpha 10 Subunit	x	x			(67)
2	CHRNB4	Cholinergic Receptor Nicotinic Beta 4 Subunit	x	x			(67)
2	DSC1	Desmocollin 1	x	x			(54, 68, 69)
2	DSC2	Desmocollin 2	x	x			(54, 68, 69)
2	HBE1	Hemoglobin Subunit Epsilon 1	x	x			(64)
2	ICAM1	Intercellular Adhesion Molecule 1	x	x			(64)
2	IGHG2	Immunoglobulin Heavy Constant Gamma 2	x	x			(64)
2	IL1RAPL2	Interleukin 1 Receptor Accessory Protein Like 2	x	x			(64)
2	IRF8	Interferon Regulatory Factor 8	x	x			(64)
2	NMNAT2	Nicotinamide Nucleotide Adenylyltransferase 2	x	x			(64)
2	PECAM1	Platelet And Endothelial Cell Adhesion Molecule 1	x	x			(64)
2	PKP3	Plakophillin 3	x	x			(70)
2	PMP22	Peripheral Myelin Protein 22	x	x			(55, 64)
1	ATP2C1	ATPase Secretory Pathway Ca2+ Transporting 1		x			(55)
1	ANXA8L1	Annexin A8 Like 1		x			(55)
1	CD1B	Cluster of Differentiation 1B molecule; Integrin beta 2		x			(55)
1	CD32	Cluster of Differentiation 32 molecule, Fc-fragment of IgG		x			(55)
1	CD88	CD88 molecule, complement component 5a receptor 1		x			(55)
1	CDH8	Cadherin 8		x			(55)
1	CDH9	Cadherin 9		x			(55)
1	CHRM4	Cholinergic Receptor Muscarinic 4		x			(63)
1	CHRNA3, –A5, A7, –A9, A10, –B2, and –B4	Cholinergic Receptor Nicotinic Subunits Alpha 3, –Alpha 5, Alpha 7, Alpha 9, Alpha 10, Beta 2 and Beta 6	x				(55)
1	CHRND	Cholinergic Receptor Nicotinic Delta Subunit		x			(55)
1	CHRNE	Cholinergic Receptor Nicotinic Epsilon Subunit		x			(55)
1	COL21A1	Collagen Type XXI Alpha 1 Chain		x			(55)
1	COLXVII	Collagen Type XVII Alpha 1 Chain	x				(71)
1	CYB5B	Cytochrome B5 Type B		x			(55)
1	DSP	Desmoplakin	x				(72)
1	ECAD	E-Cadherin	x				(73)
1	FCER1	Fc Fragment of IgE receptor 1	x				(74)
1	FH	Fumarate Hydratase		x			(55)
1	GBP1A	Glycoprotein Ibα					(55)
1	HLA-DRA	Major Histocompatibility Complex, Class II, DR Alpha		x			(55)
1	HLA-E	Major Histocompatibility Complex, Class I, E		x			(55)
1	NDUFS1	NADH:Ubiquinone Oxidoreductase Core Subunit S1		x			(55)
1	PDHA1	Pyruvate Dehydrogenase E1 Alpha 1 Subunit		x			(55)
1	SCL36A4	Solute Carrier Family 36 Member 4		x			(55)
1	SOD2	Superoxide Dismutase 2		x			(55)

*Black (level of evidence: 1) indicates that PV-relevant antigens were found by one study and/or one methodology. Green (level of evidence: 2) indicates that PV-relevant antigens were found by one or more studies and two independent methods. Red (level of evidence: 3) indicates that PV-relevant antigens were found by one or more studies and three independent methods and/or confirmed in vivo*.

Some of the first non-Dsg targets of autoAbs to be discovered were those directed against acetylcholine receptors. Using PVIgG to immunoprecipitate keratinocytes whose cholinergic receptors were first radiolabeled using [^3^H]propylbenzilylcholine mustard, it was shown that 34/40 PV patients precipitated cholinergic receptors (44). In an attempt to identify which cholinergic receptor may be recognized by autoAbs, it was shown that pre-incubation of monkey esophagus with PVIgG blocked the binding of antibodies directed at alpha9 acetylcholine receptor. Using antibodies derived from rabbits this group was able to show that these Abs had similar effects on the cell morphology of oral keratinocytes as PVIgG, but passive transfer of such antibodies was unable to induce blister formation (51).

PV autoAbs have also been shown to target mitochondrial proteins. PVIgG can penetrate keratinocytes and bind targets on the mitochondrial membrane. In one study 6/6 PV sera contained autoAbs that recognized mitochondrial preparations purified from keratinocytes, although the molecular weights of reactive proteins varied from sample to sample. Removal of these mitochondrial autoAbs by pre-incubation with mitochondrial preparations abolished the ability of PVIgG to cause acantholysis in a keratinocyte monolayer and lessened the severity of suprabasilar blister formation in a passive transfer model (75). In a separate experiment, PVIgG was also shown to precipitate various mitochondrial nicotinic cholinergic receptor subtypes. The mitochondrial nicotinic subtype α3 was precipitated by 3/5 patients, α5 by 2/5, α10 by 2/5, β2 by 1/5, and β4 by 1/5 (67).

Other studies have shown that some PV sera bind desmocollins (Dsc) 1−3. An immunoblot of bovine desmosomal preparation identified 4/16 PV sera recognizing Dsc 1/2 (68), while another study also performing immunoblot analysis identified Dsc 1/2 autoAbs in 8/39 PV patients. Constructs consisting of the extracellular domains of each Dsc isoform, however, were not recognized by these sera (69). Yet another study demonstrated that 8/39 PV samples immunoprecipitated Dsc3, and that preabsorption of sera with recombinant Dsc3 prevented the ability of this PVIgG to cause acantholysis in a cell monolayer (53). Recently, another study using ELISAs made with Dsc proteins expressed in mammalian cells found that in a group of 22 PV patients, 2/22, 3/22, and 1/22 patients were positive for autoAbs against Dsc1, 2, and 3 respectively (54).

Another keratinocyte antigen found to be detected by PVIgG was an annexin-like protein, now known as pemphaxin. To identify this protein, PVIgG was purified using the PVIg construct and eluted autoAbs that recognized a 75 kDa band were used to screen a library of keratinocyte proteins. Preabsorption of PVIgG using a recombinant version of pemphaxin eliminated the ability of PVIgG to cause blister formation when passively transferred to mice. However, autoAb eluted from this column, while able to restore acantholytic ability to previously pre-absorbed PVIgG, was not sufficient to induce blister formation in mice (52).

A number of other experiments, where identification of PV autoantigens was not the primary goal, have still provided information concerning the reactivity of autoAbs in PV. Immunoblotting PVIgG identified 3/44 pemphigus sera containing autoAbs that recognized full length collagen XVII (71). Sera from two PV patients was shown to react with a recombinant Dsg4 protein (76). In a case review, a patient with PV was shown by immunoblot to have antibodies against desmoplakin (72). Another experiment which coupled immunoprecipitation with immunoblotting identified anti-E-cadherin autoAbs in 33/40 PV patients. However, indirect immunofluorescence of A431DE cells, which express E-cadherin but not Dsg1, was negative. These results indicate that E-cadherin positivity in PV patients may be a result of cross reactivity of Dsg1 autoAbs with E-cadherin (73). Plakophilin 3 (Pkp3) reactivity was observed in 1/4 PV patients when immunoblotting against the lysate of HEK293 cells transfected with a gene encoding for Pkp3 (70). Using an ELISA specific for FcER1, it was determined that 12/28 PV patients had autoAbs directed against FcER1 (74). Several additional studies have assessed anti-thyroid peroxidase (TPO) autoAb levels and found that between 14 and 40% of PV patients have autoAbs directed against TPO (58–62).

## Protein array technology

Protein microarrays are powerful tools that allow for the assessment of protein interactions in a high-throughput manner. Compared to previous techniques such as ELISA, protein microarrays are more sensitive, require less sample volume, and can probe for multiple protein-protein interactions simultaneously, making them an especially powerful tool for assessing the autoAb response in autoimmune diseases. The use of protein arrays has facilitated the identification of novel antigenic targets in multiple autoimmune diseases, including the identification of biomarkers in RA which predate disease by months to years and specific autoAb profiles that predict disease phenotype and prognosis in polyomyositis (77).

**Figure 2:**
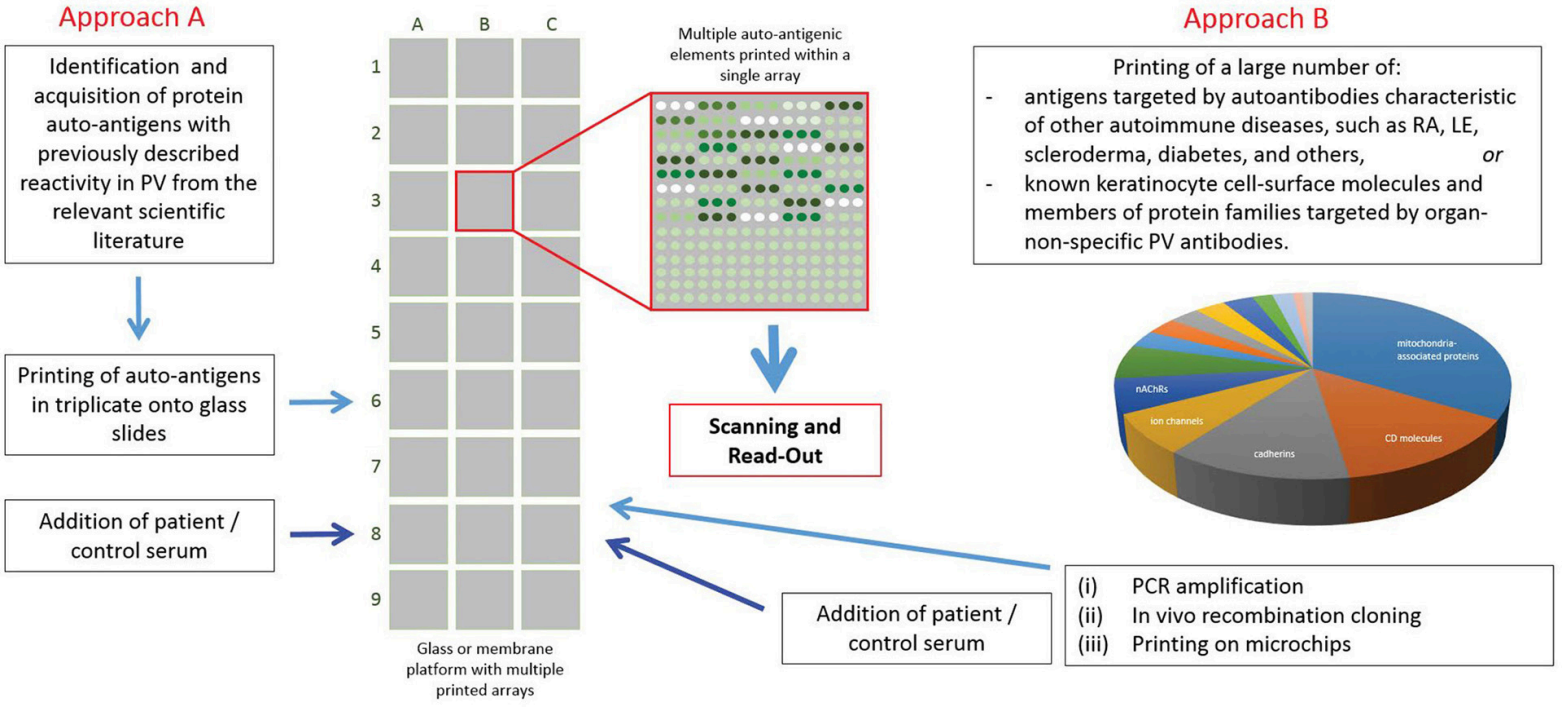
Use of protein array technology in Pemphigus vulgaris. To date, protein array technology has been used by 2 groups with differing sets of antigens printed, patient and control populations used, and varying approaches to analysis. Both groups found some overlap in the autoantibody response in PV for both anti-Dsg and non-Dsg targets.

Recently, protein array technology has been used to characterize the scope of antigens targeted by autoAbs in PV (Figure 2). Kalantari-Dehaghi et al. (64) probed autoAb reactivity of seven PV patients and five healthy controls using a protein microarray consisting of 785 keratinocyte antigens (expressed using a cell-free expression system). These authors detected 16 antigens with significantly higher reactivity in PV sera compared to healthy sera: thrombospondin receptor (CD36), immunoglobulin heavy chain constant region gamma 2 (IGHG2), TNF receptor superfamily member 5 (CD40), CD37, nicotinamide/nicotinic acid mononucleotide adenylyltransferase 2(NMNAT2), CD84, peripheral myelin protein 22 (PMP22), hemoglobin epsilon 1 (HBE1), interferon regulatory factor 8 (IRF8), CD2, carcinoembryonic antigen-related cell adhesion molecule 6 (CEACAM6), platelet/endothelial cell adhesion molecule (PECAM1), cholinergic receptor, muscarinic 1 (CHRM1), CD33, interleukin 1 receptor accessory protein-like 2 (IL1RAPL2), intercellular adhesion molecule 1 (ICAM1). These findings were then confirmed by immunoblot (64). This experiment indicated the autoAb response in PV is more complicated than initially thought, but the power of analysis was limited due to the small number of patients.

The same group later ran a similar experiment comparing the IgG autoAb reactivity of 264 PV patients with158 healthy controls. This analysis identified a large number of proteins that were targeted at least 10 fold greater by autoAbs in PV sera vs. that of controls: sialic acid-binding immunoglobulin-like lectin 3 (CD33; ratio = 27.7) and glycoprotein Iba (GP1BA; 27.7), d subunit of nicotinic AChR (CHRND; 17.6), proton-coupled amino acid transporter 4 (SLC36A4; 17.3), the antigen-presenting protein CD1B (13.1), Fc-fragment of IgG (CD32; 12.5), cadherin 8 (CDH8; 11.3) and 9 (CDH9; 11.5), peripheral myelin protein 22 (PMP22; 11.0), the MHC class I molecule E (HLA-E; 10.8) and the mitochondrial proteins NADH-ubiquinone oxidoreductase (NDUFS1; 16.2), cytochrome b5 outer mitochondrial membrane isoform precursor (CYB5B; 13.1), superoxide dismutase (SOD2) a subunit of pyruvate dehydrogenase E1 component (PDHA1; 10.3) and fumarate hydratase (FH; 10.1). The antigens that were recognized by the majority of PV patients were DR α chain of the class II major histocompatibility complex (MHC) encoded by the human leukocyte antigen (HLA)-DRA gene (45% PV patients), Dsc1 and Dsc3, respectively; 44% each), ATPase, Ca++ transporting, type 2C, member 1 (ATP2C1; 43%), plakophilin 3 (PKP3; 43%), M3 subtype of muscarinic acetylcholine receptor (AChR) (CHRM3; 42%), collagen a1, type XXI, (COL21A1; 42%), annexin A8-like 1 molecule (ANXA8L1; 42%), complement component 5a receptor 1 (CD88; 42%) and e subunit of nicotinic AChR (CHRNE; 41%) (55).

Concurrently, our group also sought to characterize autoAb reactivity (both Dsg as well as non-Dsg) in PV patients using protein microarray technology (Figure 2). In contrast to previous studies, we designed a focused, disease-specific custom protein array that included (in addition to relevant biological and technical controls): Dsg1-4, Dsc 2 and 3, CHRM1 and 3-5, Pkg, E-cad, TPO, FCER1, and ANXA9, all identified as potential targets of disease relevant autoAbs by a thorough review of the literature at the time of experimentation. Since post-translational modifications are known to affect the reactivity of autoAbs in PV (17, 78, 79), printed antigens were produced in cell-based expression systems to more closely mimic typical posttranslational modifications. Analysis of autoAb using sera from 40 active PV patients and 20 healthy controls revealed significantly increased IgG reactivity toward Dsg3, CHRM 3,4,5, and TPO in PV patients (63). Interestingly, PV patients also exhibited a significant *decrease* in IgM reactivity to the same 5 antigens compared to healthy controls, while healthy controls with no history of autoimmune disease, who were first or second degree relatives of PV patients, had *increased* IgG autoAb reactivity to these same antigens. Further investigation suggested that this IgG reactivity in healthy related controls was partially linked to the expression of specific HLA alleles (DQB01^*^0503 and DRB01^*^0402), which are known to be strongly associated with PV (80, 81). This highlights the unique ability of protein microarrays to examine how genetic elements can impact the immune response.

In a subsequent study, we utilized an expanded protein microarray encompassing a wider range of putative PV autoantigens to better characterize the autoAb response in PV and identify patterns of autoAb reactivity that might underlie disease heterogeneity (82). Using this next generation array, we performed the largest known analysis assessing IgG autoAb reactivity in PV (466 patient and 216 control samples) and identified significantly increased reactivity toward 35 Ags, including all four non-Dsg autoAgs identified in our previous array. Again, the PV associated HLA risk alleles described above were shown to impact the atoAb profiles in patients and HLA-matched healthy controls. In addition, we also identified significantly increased reactivity toward 19 Ags in patient samples obtained from patients in the active phase of disease when compared to samples obtained from patients who were in disease remission as defined by consensus definitions (83). Furthermore, by comparing autoAb reactivities from samples obtained from patients who exhibited distinct disease morphologies at the time of sample collection [either mucosal (M), cutaneous (C), or mucocutaneous, (MC)], we were able to identify distinct profiles of autoAb reactivity that correlated to disease morphology.

Additional longitudinal analyses of samples obtained from patients across various time points and in different phases of disease activity demonstrated that changes in autoAb profiles were associated with variance in disease activity in all cases. However, the precise sets of antigens recognized was unique to individual patients. Finally, using specific patterns of autoAb reactivity identified in the previous analysis, and accounting for the known history of disease morphology, we were able to accurately predict the disease activity and expression in de-identified patient samples, indicating the potential for identifying serum biomarkers with clinical utility.

Together, these data strongly support the idea that non-Dsg autoAbs underlie disease complexity in PV and, furthermore, demonstrates the utility of comprehensive autoAb profiling to accurately classify, monitor and predict disease activity.

## Functional role of non-desmoglein autoantibodies in PV

Currently, a direct and definitive functional role in PV has yet to be established for any non-Dsg autoAb. However, there is evidence suggesting a potential role in the disease process for a number of these autoAbs. Here, we assess both the methods utilized in the detection of the non-Dsg autoAbs, as well as data with implications to disease function in order to better understand how non-Dsg autoAb may be relevant to disease pathology.

### Cholinergic receptors

Epidermal keratinocytes express both nicotinic and muscarinic acetylcholine receptors, and both receptor subtypes function together in order to maintain cell-cell adhesion (84). The importance of cholinergic signaling in cell adhesion, as well as its relevance to PV, is emphasized by the ability of both: (1) muscarinic and nicotinic agonists to abolish PVIgG induced acantholysis *in vitro* and *in vivo*, and (2) muscarinic and nicotinic antagonists to induce cell separation in cultured keratinocytes (85–87). It should also be noted that these cholinergic receptors exhibit differential expression throughout the layers of the epidermis as well as between cutaneous and mucosal tissue (88), targeting of which could potentially contribute to the various disease phenotypes and characteristic level of acantholysis seen in PV.

Alteration of cholinergic signaling is also relevant in the clinical treatment of pemphigus. One study showing that PV patients who smoked cigarettes had better response to therapy and that smokers are less likely to develop PV (89–91) may implicate imbalances in nicotinic cholinergic signaling in disease pathogenesis. However, the therapeutic effects of cigarette smoking may very well be a function of the ability of nicotine to increase endogenous glucocorticoid production (92) or suppress B cell proliferation (92), rather than action on keratinocyte receptors. In another study, Mestinon, a compound which interferes with the breakdown of acetylcholine, was used to treat 6 PV patients as well as 1 patient with paraneoplastic pemphigus (PNP) and another with PF. Three of the 6 PV patients treated with this compound exhibited significant improvement (93). However, the therapeutic effects of these compounds may arise simply due to their pro-adhesive effects, and even though they may represent a therapeutic target, the observed effects alone do not indicate a clear role for anti-cholinergic receptor autoAbs in disease.

Although a direct pathologic role has yet to be established, the presence of anti-cholinergic receptor autoAbs in PV and the known functional association of cholinergic receptors to cell-cell adhesion establish their candidacy as disease relevant autoAbs. Of the cholinergic receptors identified as targets of autoAbs, investigation of the functional effects of anti-CHRM3 and anti-annexin (ANXA)9 autoAbs appear to be of highest relevance, as autoAbs targeting each were identified by two distinct approaches (55, 64, 85–87). Furthermore, the relatively large study groups used in the protein array studies (55, 64) indicate that these autoAbs are prevalent in the PV population.

### Mitochondrial proteins

Anti-mitochondrial autoAbs are found in several other autoimmune disorders in addition to PV, such as primary biliary sclerosis and systemic scleroderma (67, 75, 94–96). Despite a lack of specificity to PV, a preponderance of evidence exists that links anti-mitochondrial autoAbs to pathogenesis in PV. Characteristically, PV patients have been shown to exhibit increased levels of oxidative stress and reactive oxygen species along with abnormalities in lipid peroxidation and mitochondrial enzyme activity, all changes associated with mitochondrial dysfunction (97–102). Additionally, anti-mitochondrial autoAbs in PV have been shown to disrupt mitochondrial oxygen respiration, membrane potential across the mitochondrial membrane, and increase cellular reactive oxygen species (103). These changes are sufficient to induce apoptotic mechanisms [reviewed in (104)], which, despite some controversy, have been shown by some groups to play a role in cell-cell detachment in PV (105). A role for anti-mitochondrial autoAb induced mitochondrial dysfunction is further supported by the reduction of blister formation in mice treated with mitochondrial protective drugs: Cyclosporin A, minocycline, and nicotinamide (103). However, it is also possible that the protective effects of these compounds, particularly minocycline and cyclosporine, may be due to their well-documented immunosuppressive effects.

Studies demonstrating that anti-mitochondrial autoAbs can penetrate keratinocytes may help to explain how autoAbs targeting intracellular proteins could contribute to disease processes (75). Recently, it was discovered that the internalization of anti-mitochondrial autoAbs (and others) in keratinocytes is dependent upon the neonatal Fc receptor (FcRn). FcRn binds IgG in a) endosomes after pinocytosis of IgG, or B) in it's membrane bound form, followed by internalization of the IgG–FcRn complex. The mechanisms by which receptor bound IgG avoid degradation is not currently understood, although one explanation may be that these endosomes are trafficked directly to the mitochondria, the site of their antigenic targets (106, 107). Blocking of the FcRn was shown to ameliorate PVIgG induced acantholysis *in vitro* (108), demonstrating the potential functional significance of this pathway. Interestingly, these experiments also found non-anti-mitochondrial autoAbs internalized through the same pathway. Given that FcRn is predominantly expressed by basal keratinocytes in the epidermis (109), this unique expression pattern may shed light on the characteristic suprabasilar site of acantholysis seen in PV.

Identifying the predominant target (or targets) of anti-mitochondrial autoAbs in PV is critical. Although functional studies have demonstrated how mitochondrial disruption could contribute to blister formation, the exact antigenic targets have not been elucidated. The increased reactivity toward a number of mitochondrial proteins as determined from protein microarray data (55) is promising, but further validation is required. Similarly, although the immunoprecipitation of mitochondrial nicotinic AChRs confirmed the presence of autoAb reactivity against 4 proteins identified by protein microarray (67), the relatively small sample size tempers the conclusions that can be drawn concerning the prevalence of such autoAbs across PV patients as a group.

### Non-Dsg adhesion proteins

AutoAb in PV are also known to target a number of non-desmoglein adhesion proteins. Of those recognized, the Dsc proteins are the most similar to the Dsg proteins. Dsc1-3 represent desmosomal cadherins (in addition to the desmogleins) that are involved in cell-cell adhesion (110–114). Similar to the desmogleins, there exists a differential expression of Dsc isoforms through the layers of the epidermis, with Dsc1 having highest expression in the most superficial layers and Dsc3 expressed primarily in the suprabasilar/deep epidermis (115–119). In addition to the adhesive functions of their extracellular domains, the cytoplasmic tails of these proteins are also known to play a role in formation of the desmosomal plaque and attachment of desmosomes to the intermediate filament network (120–124). The high degree of structural and functional similarity between these two groups of proteins reflects the potential functional relevance of autoAbs directed at these targets.

Dsc3 in particular represents a good target candidate for potentially disease relevant autoAbs. Dsc3 knockout mice develop a PV-like phenotype with spontaneous suprabasilar blister formation (56). Additionally, anti-Dsc Abs can cause acantholysis in both keratinocyte monolayers as well as in a model of human epidermis (57). PVIgG pre-absorbed to remove anti-Dsc3 autoAbs is no longer pathogenic (53). The observation that greater than 40% of patients harbor anti-Dsc3 autoAbs (55) further supports the notion that Dsc3 may represent a target of disease relevant autoAbs in PV.

Plakophillins, in conjunction with plakoglobin, facilitate the attachment of the desmosomal cadherins to desmoplakin and the keratin intermediate filament network (125–128). Plakophillins have also been shown to play a key role in the assembly of desmosomes (129, 130), and mutations of plakophilins are known to cause ectodermal dysplasia-skin fragility syndrome, a disease similar to PV that is characterized by mechanical stress-induced blister formation (131). Given their role in cell adhesion, it is possible that the binding of autoAb to these targets may lead to dysfunction, resulting in impaired cell-cell adhesion. However, it has not yet been shown that PVIgG interacts with intracellular plakophillins *in vivo*.

Although autoAbs targeting other non-desmoglein adhesion associated proteins have been identified in PV patients, little functional data exists to suggest a role for these autoAbs in disease. However, evidence describing the relationship between E-cadherin and desmosomes may suggest a role for autoAbs targeting this protein in PV. E-cadherin, like the desmogleins, is a member of the cadherin family of proteins. However, unlike desmogleins, E-cadherin is known to associate with adherens junctions as opposed to desmosomes (132). While not directly involved in desmosomal adhesion, E-cadherin has been shown to play a role in the recruitment of both Dsg3 and Pkp3, suggesting a role for E-cadherin in the early stages of desmosomal development (133, 134). Given this relationship, anti-E-cadherin autoAbs identified in PV patients (73) may interfere with the normal functioning of E-cadherin, resulting in impaired desmosomal formation.

### Additional targets

Autoantibodies to a number of additional targets have been found in PV, as detailed above. Their potential functional significance in PV is explored below.

#### Thyroid peroxidase (TPO)

TPO, an enzyme that functions in the organification of iodine, is a major target of autoAbs in autoimmune thyroid disease (135). The increased risk of autoimmune thyroid disease in both PV patients and first degree relatives highlight the association of autoimmune thyroid disease and PV (58–60, 136, 137). Recently, our lab has found an increased prevalence of anti-TPO autoAbs in PV patients vs. controls that is linked to the absence of both PV-typical HLA alleles and of anti-Dsg Abs (62). In a separate study, we also show that anti-TPO Abs can induce cell fragmentation in keratinocyte dissociation assays, and affect intracellular Ca levels along with p38MAPK activation in a manner similar to anti-Dsg3 autoAbs (82). Establishing a direct pathogenic role for these autoAbs is a continuing effort. Although, TPO mRNA has been shown to be expressed by qPCR analysis of human skin biopsies (138), protein expression in keratinocytes has yet to be demonstrated. If TPO is expressed by keratinocytes, it is possible that anti-TPO autoAbs may function in a similar manner as they do in Hashimoto's thyroiditis, inflicting cell damage via compliment fixation and/or antibody dependent cell-mediated cytotoxicity (ADCC) (139–145), though the paucity of immune cell infiltrate characteristic of PV may exclude ADCC as a major contribution to disease pathogenesis.

Another possibility is that anti-TPO Abs cross-react with other, yet to be identified non-TPO keratinocyte protein. For example, anti-TPO autoAbs may exert their pathogenic effect due to cross reactivity of these autoAbs with heat shock protein 60 (Hsp60), a mitochondria chaperone (146). Anti-Hsp60 autoAbs have been associated with a multitude of autoimmune diseases (147–151). Furthermore, these autoAbs have also been shown to reduce mitochondrial activity and activate caspase 3 (152). Additional cross reactivity observed between anti-Hsp60 Abs and acetylcholine receptors (153, 154) may also suggest that anti-TPO autoAbs could interfere with cholinergic signaling in the skin. Additionally, the selectivity of HLA-DR expressing APCs to activate T cells through Hsp60 presentation may offer an intriguing insight into the mechanisms underlying the genetic susceptibility seen in PV patients expressing the HLA DRB^*^0402 allele (155). Although it is clear that the precise mechanisms need to be worked out, the efficacy of Hsp60 tolerization in treating autoimmune conditions in both mice and humans (156–158) may represent a novel therapeutic approach to PV treatment.

#### Peripheral myelin protein 22 (PMP22)

Autoreactivity to both peripheral myelin protein (PMP)22 and CD33 was noted by Kalantari-Dehaghi et al. to be expressed at levels 10x or greater in active patients vs. controls (55). CD33 represents a transmembrane sialic acid receptor expressed on both myeloid and lymphoid cell, with no clear relationship to PV. PMP22, on the other hand, is an integral membrane protein structurally related to Perp (also seen by protein microarray to be recognized by 31% autoAbs of PV patients and only 5% of healthy controls) (55). Perp is associated with desmosomes and is integral to cell-cell adhesion (159). Deletion of Perp in mice leads to the disruption of desmosomes and spontaneous blister formation (160), and is also known to activate the extrinsic apoptotic pathway via caspase 8 activation (161). Although both Perp and PMP22 are in the same protein family, little is known about PMP22. Mutations in PMP22 are associated with Charcot Marie Tooth disease (162). PMP22 mRNA is expressed highly in all ectodermal tissues, including the skin, and staining of the MDCK cell line reveals that PMP22 localizes to areas of cell-cell contact in epithelial monolayers (163, 164). While there is no mention of epidermal alterations in any of the mouse models lacking PMP22 (165), recent studies have shown that PMP22 may play a role in anchoring the actin cytoskeleton to the plasma membrane (166). More studies ascertaining the function of PMP22 in the epidermis are needed before we can speculate on a potential role in PV.

#### Human leukocyte antigen (HLA) proteins

Expression of certain HLA-DR and HLA-E alleles is associated with susceptibility to PV (80, 167). Interestingly, antibodies to both HLA-DR and anti-HLA-E antibodies may play a role in PV pathogenesis as well. HLA-DR is expressed in low levels on basal keratinocytes, and studies have shown that expression of HLA-DR is elevated in both lesional and non-lesional skin in PV (168, 169). HLA-E expression has not been previously associated with PV skin, but keratinocytes near blisters in Stevens Johnson's Syndrome have been shown to increase expression of HLA-E, which enhances the chances of cell death by NK T cells, who require the atypical class I HLA-E molecule to be primed (170). Our group has additionally shown increased HLA-E expression in Dsg specific T-cells in the peripheral blood of patients (unpublished data). Finally, anti-HLA autoAbs have been shown to be pathogenic in pemphigoid gestations, another autoimmune skin blistering disease (171).

#### Calcium transporting ATPase type 2C (ATP2C1)

Calcium transporting ATPase type 2C (ATP2C1) encodes for a calcium pump typically located in the Golgi apparatus. This calcium ATPase represents a particularly interesting putative target for PV autoAbs because genetic mutations in this pump are known to cause Hailey-Hailey disease (172), which manifests as a loss of epidermal adhesion at the same level of the epidermis as PV. Additionally, alterations in intracellular calcium, which underlie pathogenesis in Hailey- Hailey disease, are also implicated in the pathogenesis of PV (173).

## Evolving concepts in PV: development of the “super-compensation” hypothesis

Just as the discovery of anti-Dsg autoAbs guided the formation of the monopathic view of PV pathogenesis, the elucidation of additional autoantigenic targets has spurred the metamorphosis of understanding toward a more comprehensive and complex model that is better equipped to explain the more subtle nuances seen in PV. This shift in how PV pathogenesis is viewed is epitomized by the development of the “Multiple Hit Hypothesis” (174). According to this theory, blister formation in PV occurs from a synergistic effect of autoAbs targeting multiple keratinocyte antigens. In the past, the relative lack of data pertaining to the scope and specificity of autoAbs in the population of PV patients and tools which could quickly and efficiently determine autoAb targets limited the ability to test this hypothesis. However, the advent of protein array technology and a greater understanding of relevant antigenic targets in PV has facilitated the dissection of the complex relationship between autoAb expression and disease phenotype.

Expanding the current view of disease pathology in PV also has considerable implications concerning the framework for assessing the underlying disease mechanisms. Alterations in numerous signaling pathways have been associated with the binding of PVIgG to keratinocyte antigens, including: PLC, PKC, Cdk2, p38MAPK, EGFR, Src, JNK, MMP-9, c-myc, GSKbeta, Fas/FasL, p53, BAX, and caspases 1,3, and 8 (75, 173, 175–186). Compared to the monopathic view, incorporation of multiple disease relevant autoAbs could allow for a more precise integration of these pathways, where specific autoAbs may alter specific pathways.

**Figure 3:**
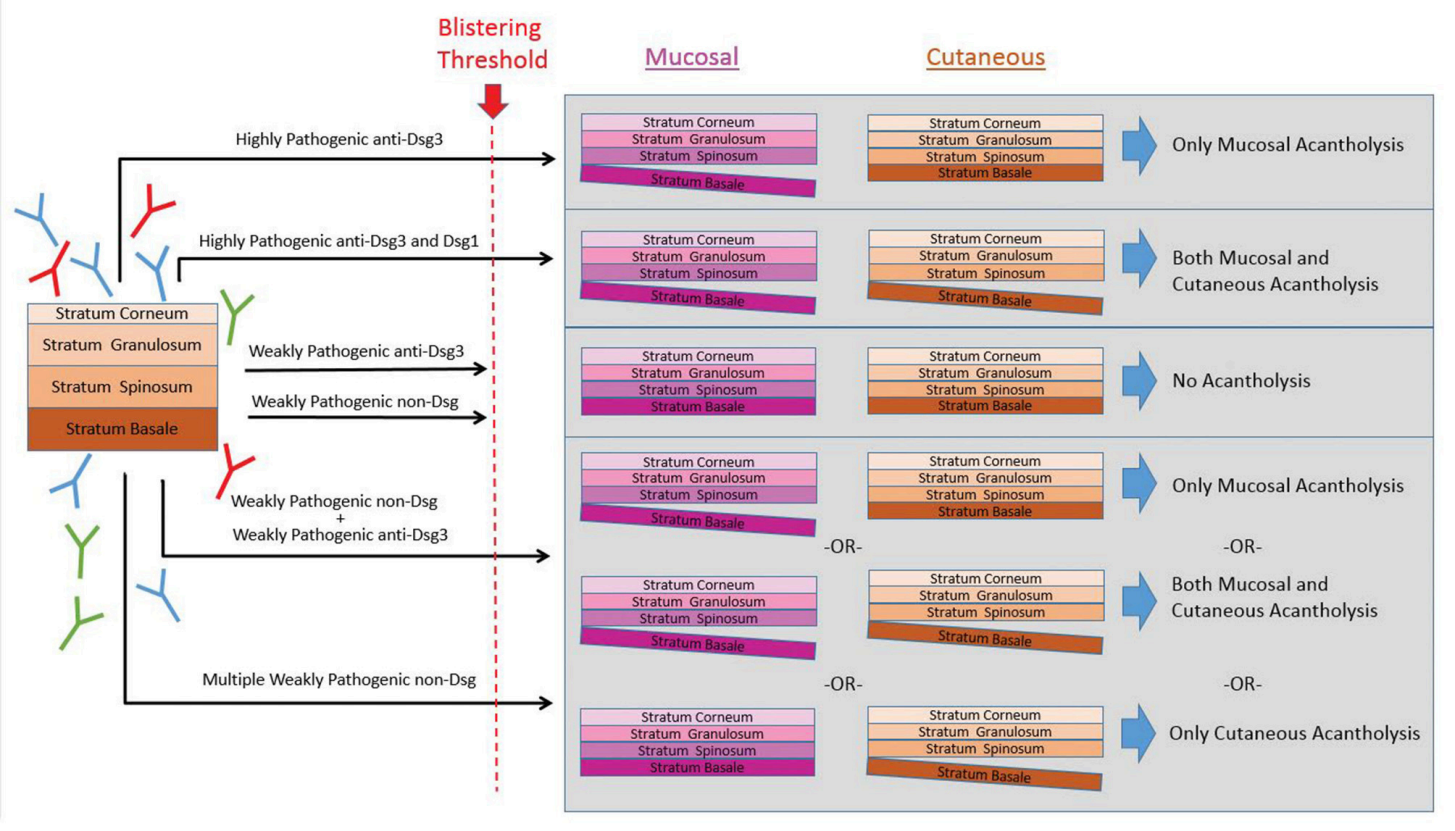
The super-compensation hypothesis. We hypothesize the binding of specific autoAbs in combination with the unique epidermal expression of the various autoantigens results in the characteristic alteration of signaling pathways and the development of acantholysis only if the *sum* of these effects exceeds a set threshold. In this theory, highly pathogenic antibodies to either anti-Dsg3 alone, or anti-Dsg3 and−1 together can exceed the blistering threshold. Similarly, multiple combinations of subpathogenic anti-Dsg3/1 autoAbs together with non-Dsg autoAbs could potentially exceed this threshold. However, weakly pathogenic anti-Dsg or non-Dsg autoAbs alone, or sometimes even in combination, do not breach the threshold for triggering acantholysis.

In consideration of the data reviewed in this manuscript, we propose a “*super-compensation hypothesis*” that purports that the binding of specific autoAbs in combination with the unique epidermal expression of the various autoantigens results in the characteristic alteration of signaling pathways and the development of acantholysis only if the *sum* of these effects exceeds a set threshold (Figure 3). Weakly pathogenic autoAbs alone, or in combination do not trigger these effects. However, highly pathogenic autoantibodies alone, or multiple combinations of pathogenic or subpathogenic autoAbs could potentially exceed this threshold (Figure 3). Furthermore, specific autoAb expression profiles may underlie variations in disease expression to better explain clinical heterogeneity across phenotypic subgroups. The role of multiple autoAbs in PV has been previously postulated (174). Here, we extend this line of thought based on the accumulating evidence from the literature and our lab presented above to formulate a novel hypothesis underlying autoAb-mediated acantholysis. This model of PV has the potential to integrate autoAb profiles, disease variability and the mechanistic effect of autoAbs in a way that was impossible to achieve when viewing PV as the result of strictly anti-Dsg autoAbs. Consequently, each of the autoantibodies potentially involved in PV pathogenesis would lead to activation of specific downstream signaling pathways that either result in pathway amplification and/or in additive/combinatorial effects relevant to acantholysis [see (186) for a comprehensive review of autoantibody signaling in PV].

Recent work by our group assessing the functional capacity of anti-TPO autoAbs present in patient IgG provides support for the idea that multiple autoAb specificities may function together to contribute to disease. By depleting PVIgG of anti-TPO autoAbs, we were able to demonstrate that anti-TPO autoAbs contributed to PVIgG induced loss of cell adhesion, as well as PVIgG induced activation of p38MAPK and increases in intracellular calcium *in vitro* (187). These results demonstrate that additional, non-Dsg autoAbs contribute to PVIgG induced pathogenesis. However, these experiments also provide key insights concerning how multiple autoAb specificities may be working together in unique ways to drive blister formation in PV. Specifically, the effects of anti-TPO depletion were most significant when anti-Dsg3 autoAbs were not present. This could potentially help to explain why some patients who are negative for both anti-Dsg3 and anti-Dsg1 still exhibit disease activity. Interestingly, in support of this notion, we find the highest prevalence of anti-TPO Abs in the subgroup of patients that lack anti-Dsg Abs (62). Additional experiments investigating the precise effects of multiple autoAb specificities are required to more fully characterize how different autoAbs function together to elicit blister formation in PV.

Additionally, researchers should consider that the role of autoAbs is not always restricted to driving pathogenesis. Natural autoAbs of the IgM subgroup have been shown to play a number of beneficial roles, with subsets of these autoAbs modulating disease severity, and even protecting against the development of in autoimmune disease (188–190). It is entirely possible that some of the autoantibodies found in PV are protective against disease, similar to the role of certain g-protein coupled receptors, such as CXCR4, in experimental autoimmune encephalomyelitis (191).

## Future directions

Ultimately, the primary objective of investigation into PV is to identify avenues of intervention to improve patient quality of life. With our current understanding of disease, the best available treatments remain the administration of glucocorticoids or other broadly immunosuppressive agents, which by themselves pose a significant risk to patient health. The lack of actionable biomarkers to monitor disease progression, response to therapy, or help predict prognosis makes it almost impossible for physicians to maximize treatment efficacy while minimizing harmful side effects.

Recent characterization of autoAb specificity represents a significant step toward achieving a broader understanding of PV. However, these results must first be validated and the autoAb repertoire of even larger patient cohorts must be assessed in order to have an accurate estimation of auto antigenic targets across all PV patients. Given the well documented importance of conformation and post-translational modifications on the immunogenicity of proteins, subsequent experiments should also be conducted using antigens produced in cell systems that will parallel those of human keratinocytes. Once the full repertoire of autoAb specificity is clear, the effects of these autoAbs on keratinocyte adhesion and any effects on cellular signaling must be ascertained.

The foundation for the significance of this proposed work lies on the identification of autoAb signatures capable of distinguishing the phenotypic variations seen in PV. To this end, our group has taken the approach to define highly specific disease subgroups stratified by both variable characteristics (disease activity, morphology, treatment, and disease duration) and static characteristics (age of onset, sex, HLA type). Establishing specific immunoprofiles for these groups will significantly impact the clinical treatment of PV. We expect that a more in depth understanding of disease relevant autoAbs will: (1) facilitate the identification of actionable biomarkers, allowing for a more precise classification of disease while simultaneously enabling physicians to predict disease progression and response to therapy, (2) provide new insights into the mechanistic pathways responsible for acantholysis, facilitating the identification of novel therapeutic targets, and (3) allow for a higher degree of personalized medicine where autoAb profiles dictate treatments individualized toward a specific patient.

## Author contributions

All authors listed have made a substantial, direct and intellectual contribution to the work, and approved it for publication.

### Conflict of interest statement

The authors declare that the research was conducted in the absence of any commercial or financial relationships that could be construed as a potential conflict of interest. The reviewer AR and the handling Editor declared their shared affiliation.
